# Identification of novel loci associated with maturity and yield traits in early maturity soybean plant introduction lines

**DOI:** 10.1186/s12864-018-4558-4

**Published:** 2018-03-01

**Authors:** Tanya R. Copley, Marc-Olivier Duceppe, Louise S. O’Donoughue

**Affiliations:** 1 Centre de recherche sur les grains (CÉROM), Inc., 740 chemin Trudeau, St-Mathieu-de-Beloeil, Québec J3G 0E2 Canada; 2 Canadian Food Inspection Agency, 3851 Fallowfield Road, Nepean, ON K2H 8P9 Canada

**Keywords:** Days to flowering, Days to pod filling, Early maturity, Genome-wide association analysis, Novel loci, 100 seed weight, Soybean (*Glycine max* (L.) Merr.), Yield

## Abstract

**Background:**

To continue to meet the increasing demands of soybean worldwide, it is crucial to identify key genes regulating flowering and maturity to expand the cultivated regions into short season areas. Although four soybean genes have been successfully utilized in early maturity breeding programs, new genes governing maturity are continuously being identified suggesting that there remains as yet undiscovered loci governing agronomic traits of interest. The objective of this study was to identify novel loci and genes involved in a diverse set of early soybean maturity using genome-wide association (GWA) analyses to identify loci governing days to maturity (DTM), flowering (DTF) and pod filling (DTPF), as well as yield and 100 seed weight in Canadian environments. To do so, soybean plant introduction lines varying significantly for maturity, but classified as early varieties, were used. Plants were phenotyped for the five agronomic traits for five site-years and GWA approaches used to identify candidate loci and genes affecting each trait.

**Results:**

Genotyping using genotyping-by-sequencing and microarray methods identified 67,594 single nucleotide polymorphisms, of which 31,283 had a linkage disequilibrium < 1 and minor allele frequency > 0.05 and were used for GWA analyses. A total of 9, 6, 4, 5 and 2 loci were detected for GWA analyses for DTM, DTF, DTPF, 100 seed weight and yield, respectively. Regions of interest, including a region surrounding the *E1* gene for flowering and maturity, and several novel loci, were identified, with several loci having pleiotropic effects. Novel loci affecting maturity were identified on chromosomes five and 13 and reduced maturity by 7.2 and 3.3 days, respectively. Novel loci for maturity and flowering contained genes orthologous to known Arabidopsis flowering genes, while loci affecting yield and 100 seed weight contained genes known to cause dwarfism.

**Conclusions:**

This study demonstrated substantial variation in soybean agronomic traits of interest, including maturity and flowering dates as well as yield, and the utility of GWA analyses in identifying novel genetic factors underlying important agronomic traits. The loci and candidate genes identified serve as promising targets for future studies examining the mechanisms underlying the related soybean traits.

**Electronic supplementary material:**

The online version of this article (10.1186/s12864-018-4558-4) contains supplementary material, which is available to authorized users.

## Background

Due to their sedentary life style and somewhat limited means of dispersal, plants have become highly attuned to their surrounding environment. Photoperiod responses are well known to impose limitations on plants such as the transition from the vegetative to flowering stage [1]. As such, plants have been grouped into short day, long day and day neutral plants depending on the number of daylight hours required to induce flowering. In addition to photoperiodism, transition from the vegetative to flowering stage is also affected by temperature (vernalisation), gibberellin hormones, as well as other factors that are not yet fully understood (example abiotic stresses) making this transition a complicated event [1]. In order to expand the growing range of short day crops, such as soybean, to far north and south regions, agricultural breeding programs have aimed to alter photoperiod responses to obtain day neutral responses.

Soybean, *Glycine max* (L.) Merr., is a short day flowering crop originating from Asia. Its grain is used worldwide as a human and animal food source, and for the production of oils and plastics. World soybean production was 320.2 M metric tons in 2015, which represented a 47% increase worldwide since 2005, and accounted for 29.0% of the world’s vegetable oil consumption and 70.9% of the protein meal consumption in 2015 (www.soystats.com). The expansion of soybean cultivation has been challenged by long days and short growing seasons of far northern/southern climates and cultivars that have been bred are often limited to a very narrow range of latitudes [2]. In order to continue to meet the increasing demands of soybean worldwide, it is crucial to identify key genes regulating flowering and maturity to expand the area of cultivated regions.

Several natural genetic variants controlling flowering and maturity time have been identified and heavily used in soybean breeding programs for adaptation to long day environments. These include the soybean *E* genes *E1* to *E10* [3–12], the *JUVENILE* (*J*) gene [13], and the *FLOWEING LOCUS T* (*FT*) genes [14]. Among the *E* genes, *E1* to *E4, E9* and *E10* have been identified by various fine-mapping and candidate gene identification approaches [6, 7, 9–12]. *E1* contains a putative bipartite nuclear localization signal and a B3-related domain [10] and acts as a floral repressor by down-regulating *GmFT2a* and *GmFT5a* [15]*.* The *E2* gene is an orthologue to the Arabidopsis *GIGANTEA* (*GI*) gene [9]; however, unlike in Arabidopsis, *E2* delays flowering under long days by inhibiting expression of *GmFT2a,* but not *GmFT5a* [9]. *E3* and *E4* encode the *PHYTOCHROME A* (*PHYA*) genes [6, 7], *GmPHYA3* and *GmPHY2*, and control flowering under high and low red to far-red ratios, respectively [16]. Loss-of-function alleles exist for *E1* to *E4* and lead to photoperiod insensitivity by allowing higher expression levels of the florigen *FT* genes and promoting flowering under long day conditions. Recently, *E10* was identified as *FT4*, which has been shown to be up-regulated by and act down-stream of *E1* [11]. Samanfar et al. [11] demonstrated that the *e10* haplotype promotes early flowering; however, this haplotype appears to be rare and the mechanisms by which it promotes flowering are not yet known. The remaining *E* genes result in early flowering under long day conditions and the genes encoding them have yet to be identified with the exception of *E9,* which was recently identified as the *GmFT2a* gene [12]. *E9* and *GmFT5a* have been shown to have redundant roles in soybean and control flowering by inducing the expression of flower-initiating genes [14].

Despite the increasing knowledge and continuous identification of genes involved in soybean flowering, there remains a plethora of missing links as well as a need for identification of new soybean genes for flowering and maturity. Soybean flowering and maturity are often positively correlated with seed yield [8], but are not correlated with 100 seed weight [8, 17, 18], two agronomical traits that are important for meeting soybean demands and maintaining quality standards for soy food products. As such, it is important to select early maturing varieties that minimally affect yield and seed weight. Genome-wide association (GWA) analyses enable the detection of genetic differences accounting for the observed variation in phenotypes. Studies have demonstrated that GWA studies containing few individuals (< 100) can successfully identify single nucleotide polymorphisms (SNPs) affecting phenotypes if the population is sufficiently genetically diverse and population structure is accounted for [19]. This study aimed to identify new loci governing early flowering, maturity and pod filling time, as well as seed weight and yield in Canadian environments using genotyping-by-sequencing (GBS), microarray and GWA analysis approaches in a diverse set of 86 soybean genotypes belonging to early maturity groups 00 to 000. Although previous studies have reported on SNPs affecting these traits in early maturity groups 0 and 00 [20], none have been reported on markers affecting these phenotypes in Canadian environments. This study identified major markers affecting phenotypic traits of interest by identifying over 67,000 SNPs that were subsequently used in GWA analysis approaches. The successful identification of novel loci, some of which contain genes known to govern the traits of interest in other model plant species, but not yet reported in soybean were identified.

## Methods

### Plant materials and phenotyping

A total of 86 soybean plant introduction (PI) lines belonging to early maturity groups 000 to 00 and having diverse geographical origins (Europe, China, Japan, North Korea, Russia, and North America) were obtained from the Germplasm Resources Information Network (www.ars-grin.gov) and were used for phenotypic and genetic evaluation (Additional file 1). Soybean lines were phenotyped for days to flowering (DTF), days to pod filling (DTPF), days to maturity (DTM), yield (kg/ha) and 100 seed weight (SW) for two (2012 and 2013) and 3 years (2011 to 2013) at sites located in Québec City and Saint-Mathieu-de-Beloeil, Québec, Canada, respectively, for a total of five site-years. At each site, soybean lines were planted in single row (2011) or two row (2012 and 2013) plots using a Modified Augmented Design [21, 22]. Phenotypes for each line were calculated as follows: DTF as the day of planting to the day at which 75% of the genotype was flowering; DTM as the day of planting to the day at which 95% of the pods within the genotype were at physiological maturity; DTPF as the number of days from DTF to DTM; yield as the grain mass per plot adjusted for population and converted to kg ha^− 1^; and 100 seed weight was taken as the average of two measurements per plot. Phenotypic data distribution and Spearman’s pairwise correlation coefficients were calculated for all trait comparisons using R version 3.3.1 [23]. All trait data were normally distributed.

### Genotyping

Sample preparation for GBS was performed as described in Tardivel et al. [24]. Briefly, soybean DNA was extracted using the Qiagen DNeasy 96 Plant Kit (Qiagen, Toronto, Canada) from 100 mg (wet weight) of soybean tissue obtained from a unique plant for each line following the manufacturer’s protocols. Libraries were prepared at the Plate-Forme d’Analyses Génomiques (Université Laval, Québec City, Canada) as described in Elshire et al. [25] using the *ApeKI* restriction enzyme. Sequencing was performed as single-end 100 bp reads on an Illumina HiSeq2000 System at the Genome Québec Innovation Centre (McGill University, Montreal, Canada), as part of a larger project, with 96 genotypes per sequencing lane.

Illumina sequence read data were processed using multiple publically available software tools in an in-house script (unpublished) similar to that reported by Torkamaneh and Belzile [26]. Briefly, adapters and barcodes were removed from reads using Trimmomatic v. 0.33 using ILLUMINACLIP <adapter file>:2:30:15, LEADING and TRAILING removal of three, SLIDINGWINDOW:3:20, and finally MINLEN:32. Sequences were then aligned to the soybean reference genome version 2 (NCBI assembly #GCA_000004515.3) using Burrows-Wheeler Alignment (BWA version 0.7.12-r1039) with the options –a –M –R followed by variant calling with SAMtools version 1.2.1 [27], sambamba version 0.6.4 [28] and bcftools version 1.2.1 [29]. BAM files were then pooled and variants filtered with vcftools version 0.1.15 [30] to maintain only biallelic sites with an overall mapping quality > 30, a read depth > 2, and present in a minimum of 20% of the genotypes. Missing genotypes were then self-imputed with Beagle version 4.1 [31, 32] using 10 iterations. In addition to performing GBS, data for the SoySNP50K microarray were obtained from the SoyBase database (https://www.soybase.org) [33] for each genotype. Microarray and GBS data were merged using an in house developed script in order to maximize the number of SNPs in the data set, with priority given to the microarray genotypes for common SNPs as this data was not imputed.

### Genetic analyses

Population structure was modeled using fastStructure version 1.0 [34] with a simple prior and 1000 iterations for cross-validation of population structure (K) ranging from one to ten. To diminish the effect of high admixture within the population, structure analysis was performed on genotypes with linkage disequilibrium (LD) < 0.9 and minor allele frequency (MAF) > 0.05 [35]. The optimal range of K was determined based on model complexity using the marginal likelihood method using the fastStructure script chooseK.py, as well as on visualization of the log marginal likelihood, cross-validation error and population visualization using Distruct version 1.1 [36]. Genetic relationships were investigated using unrooted Neighbour Joining (NJ) tree construction implemented in TASSEL software version 5.2.17 [37].

Linkage disequilibrium (LD), estimated as the r^2^ between SNPs, was calculated for each chromosome based on the entire collection for 10 Mb windows using Plink version 1.90b3z [38]. LD was visualized using the mean r^2^ within bin sizes of 1000 SNPs for each chromosome. LD decay was calculated as the point at which the chromosomes reached 50% of their original LD value.

SNP distributions for the GBS, microarray and merged data sets were visualized using Circos version 0.67-7 [39] ideogram and karyotype options. The effects of SNPs within the genome were calculated using SnpEff version 4.3i [40]. Locations of genes were obtained from SoyBase GFF3 files for each chromosome, converted to a GTF file using the Cufflinks version 2.1.1 command gffread [41], and a database for version 2 of the soybean genome and transcriptome built using the SnpEff build –gtf22 command.

### Genome-wide association analyses

Genome-wide association analyses were performed using TASSEL software version 5.2.17 [37]. General linear models (GLM) were performed with or without covariates from principle component analyses (PCA) or population structure covariates (*Q* = 3) obtained from fastStructure. A range of principle components (*P*) were used to reflect the chosen population structure (*P* = 3) or to represent approximately 50% of the total variation within the data set (*P* = 10). A kinship matrix was calculated for each data set using the scaled identity-by-state (IBS) method [42] implemented in TASSEL version 5.2.17 to determine the relatedness among the individuals. Compressed mixed linear models (CMLM) incorporating the kinship matrix as a random effect, along with the abovementioned principle components or population structure were tested. All GWA analyses were performed with MAF ≥0.05 and LD < 1, as well as with year and site as fixed effects. The fit of the different models for each trait was assessed by comparing the expected versus obtained –Log10 *P*-values (i.e. QQ-plots) with graphs produced in R [23]. SNPs were identified as significant in the best-fit model using Bonferroni multiple comparisons correction and visualized using the QQman package [43] in R software version 3.3.1 [23]. Significant markers within the same genomic region and in high LD were viewed as a quantitative trait locus (QTL). Candidate genes associated with SNPs were reported by calling genes within 500 kb of significant SNPs [44]. Statistical validation of SNP markers identified in the GWA analysis was done using Tukey’s HSD tests.

## Results

### Distribution and correlation of phenotypic traits

**Fig. 1:**
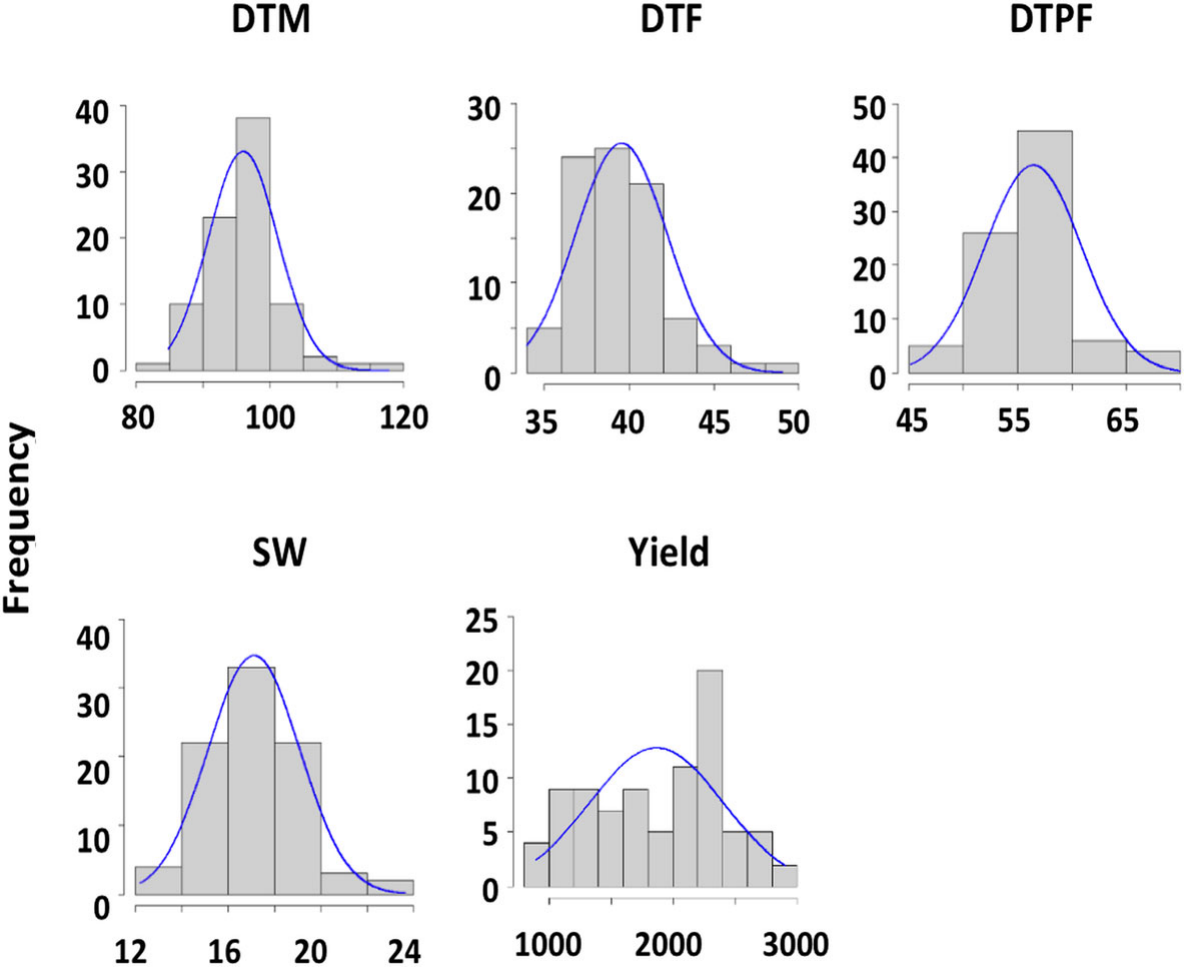
Frequency distribution of soybean traits of interest. Frequency distributions are based on the average phenotype value of each soybean line across different environments and years

Phenotyping was conducted at two different sites for two or 3 years to study the distribution and correlation among the different traits of interest. Differences among sites and years were observed among traits, and as such sites and years were used as fixed effects for all linear models (Additional file 2). Normal distributions without any significant skewness were observed for all traits (Fig. 1 and Additional file 3). Coefficients of variation ranged from 10 to 18% for all traits except yield, which was highly variable among years and environments with a coefficient of variation of 47%.

Significant correlations were observed between traits (Table 1). Highly significant correlations between DTM and DTF (*r* = 0.78) and DTM and DTPF (*r* = 0.75) were observed suggesting that later maturity may be partially due to later flowering and longer pod filling times. Similarly, 100 seed weight had a moderate negative correlation with DTF suggesting possible interdependence, while a low negative correlation was observed with DTM.

**Table 1:** Spearman’s correlation coefficients between the different traits of interest

	DTF	DTPF	Yield	SW
DTM	0.78**	0.74**	0.68**	−0.43**
DTF		0.25**	0.37**	−0.62**
DTPF			0.62**	−0.00
Yield				−0.13*

Stars represent significant differences where *P < 0.05 and **P < 0.01

DTM, days to maturity; DTF, days to flowering; DTPF, days to pod filling; SW, 100 seed weight; Yield (kg ha^− 1^)

### Distribution of SNPs and comparison of different genotyping methods

Sequencing of the GBS libraries resulted in approximately 202.2 M clean reads with an average of 2.35 M clean reads per soybean genotype with an average depth of coverage of 4.4 ± 2.2. By applying several in house filtering parameters, a total of 33,575 SNPs and 3236 INDELs were obtained. SNPs and INDELs were located throughout the genome with 69 SNP intervals over 500 kb (Additional file 4). An additional four SNPs were located in plastid DNA, with 18 SNPs and 12 INDELs in mitochondrial DNA.

**Fig. 2:**
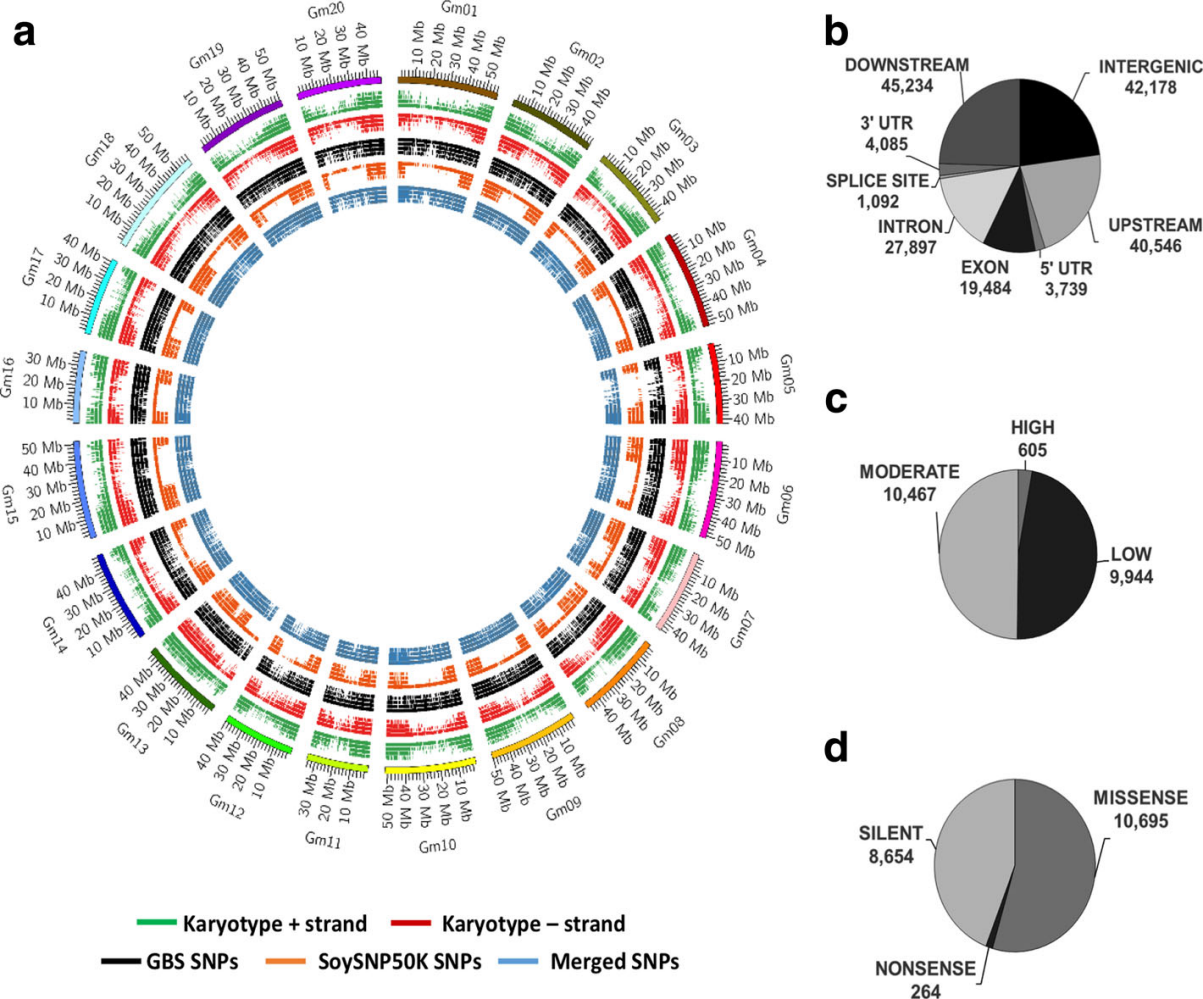
SNP distributions across the soybean genome (v2) and SNP effects within the population of plant introduction genotypes. **a** Gene and SNP distributions used for genotyping across the soybean chromosomes. From the outer to inner circle: Soybean chromosomes 1 to 20; gene locations on the positive and negative chromosome strands; and GBS, SoySNP50K microarray and the merged data set SNP locations. **b** Distribution of SNPs based on genomic region within the merged data set. **c** Predicted SNP effects based on degree of impact within the merged data set. **d** Predicted SNP effects based on function class for SNPs located within coding regions within the merged data set

Compared to the SoySNP50K microarray SNP calls, which contained a total of 34,556 SNPs, the distribution of SNPs within the GBS data set was more uniform across chromosomes, whereas SNPs within the SoySNP50K microarray data set were denser around chromosome ends (Fig. 2a). Merging of the two data sets showed that only 471 SNPs were found in both the GBS and SoySNP50K microarray data sets resulting in a total of 67,571 SNPs. Of the overlapping SNPs, a concordance rate of 98.8 ± 0.3% was observed, suggesting high reproducibility between the two data sets for common SNPs. Of the non-concordant SNPs, 62.6% were observed to be heterozygous in one of the two data sets, but homozygous in the other. Merging of the two data sets provided greater genome coverage with SNPs being relatively evenly distributed throughout the entire genome (Fig. 2a). Merging of the data sets resulted in an average SNP spacing of 13,249 bp compared to one every 28,099 bp in the GBS data set. A total of 41 gaps over 500 kb (Additional file 5) was observed in the merged data set. All gaps over 500 kb were found in centromeric or pericentromeric regions (Additional file 5). As such, this data set was used for all further analyses.

Analysis with SnpEff showed that SNPs in the merged data set were found primarily outside of coding regions, while 30.6% of SNP effects were found within genes (Fig. 2b). A total of 605 SNPs were predicted to have a high impact on gene products (Fig. 2c), while 54.5%, 1.4% and 44.1% of SNP effects within coding regions resulted in missense, nonsense and silent mutations, respectively (Fig. 2d). An overall transition to transversion ratio of 2.63 was observed.

### Population structure and linkage disequilibrium

**Fig. 3:**
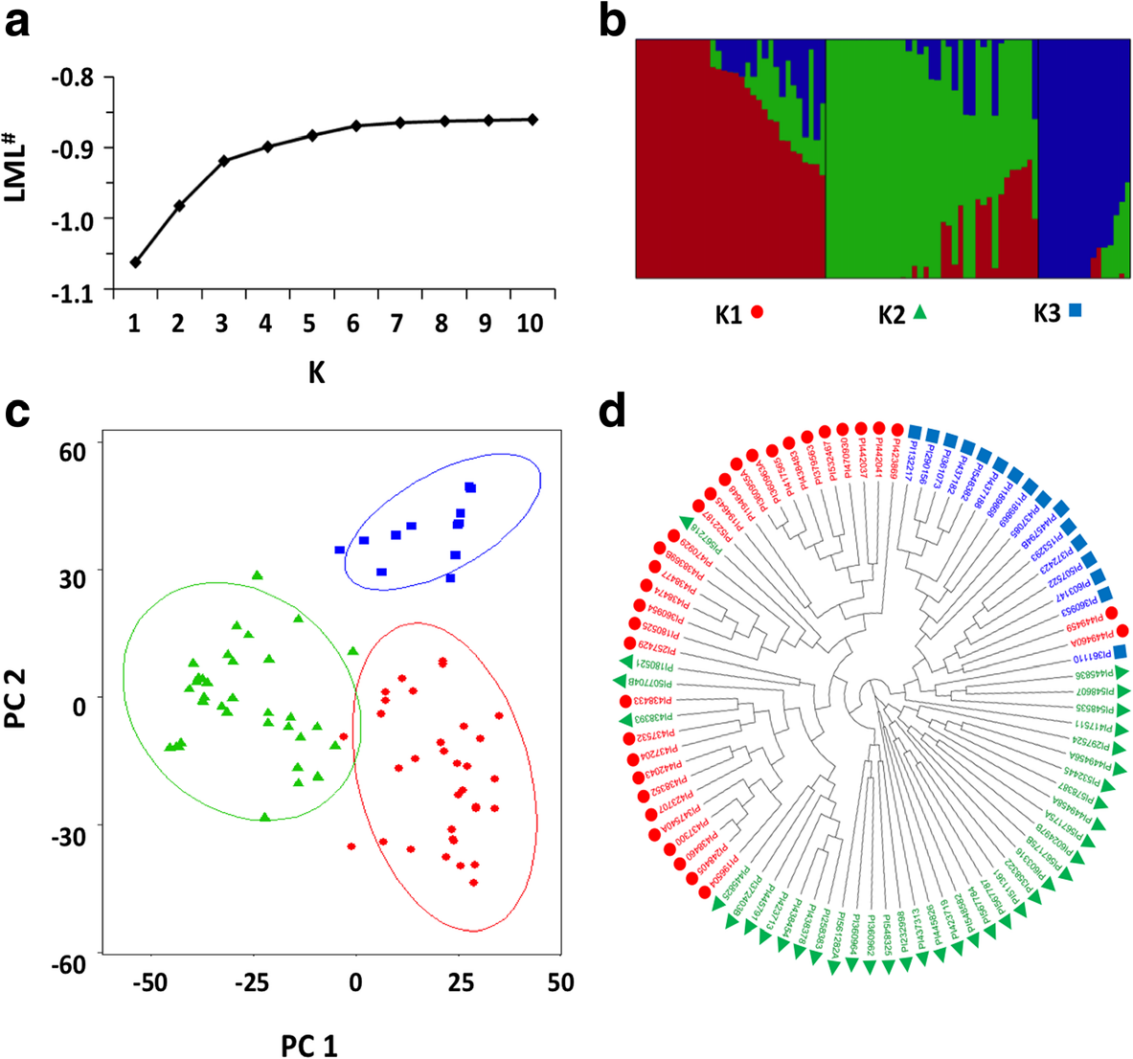
Genetic diversity and population structure of the 86 soybean genotypes. **a** Estimated log marginal likelihood (LML) calculated for populations (*K*) ranging from 2 to 10 using fastStructure. **b** Population structure of the soybean lines, where each vertical line represents a cultivar and each colour a separate population. **c** PCA plot of the first two principle components based on genotypes. Ellipses represent Hotelling’s T^2^ 95% confidence intervals for each population. **d** Cladogram of the soybean lines constructed using the neighbour-joining method. Different populations as determined by LML are indicated by identical symbols (triangle, circle and square) in all panels

Population structure analysis with fastStructure suggested between one and nine populations among the 86 plant introduction genotypes used in this study. Further analysis of the data revealed that plateauing of the marginal likelihood values started occurring at three populations (Fig. 3a, b), which is in accordance with analyses using Structure (data not shown). Principle component analysis confirmed that high similarity existed within the three populations (Fig. 3c). Neighbour joining tree analysis further confirmed the presence of three main populations and the nine subpopulations (Fig. 3d).

**Fig. 4:**
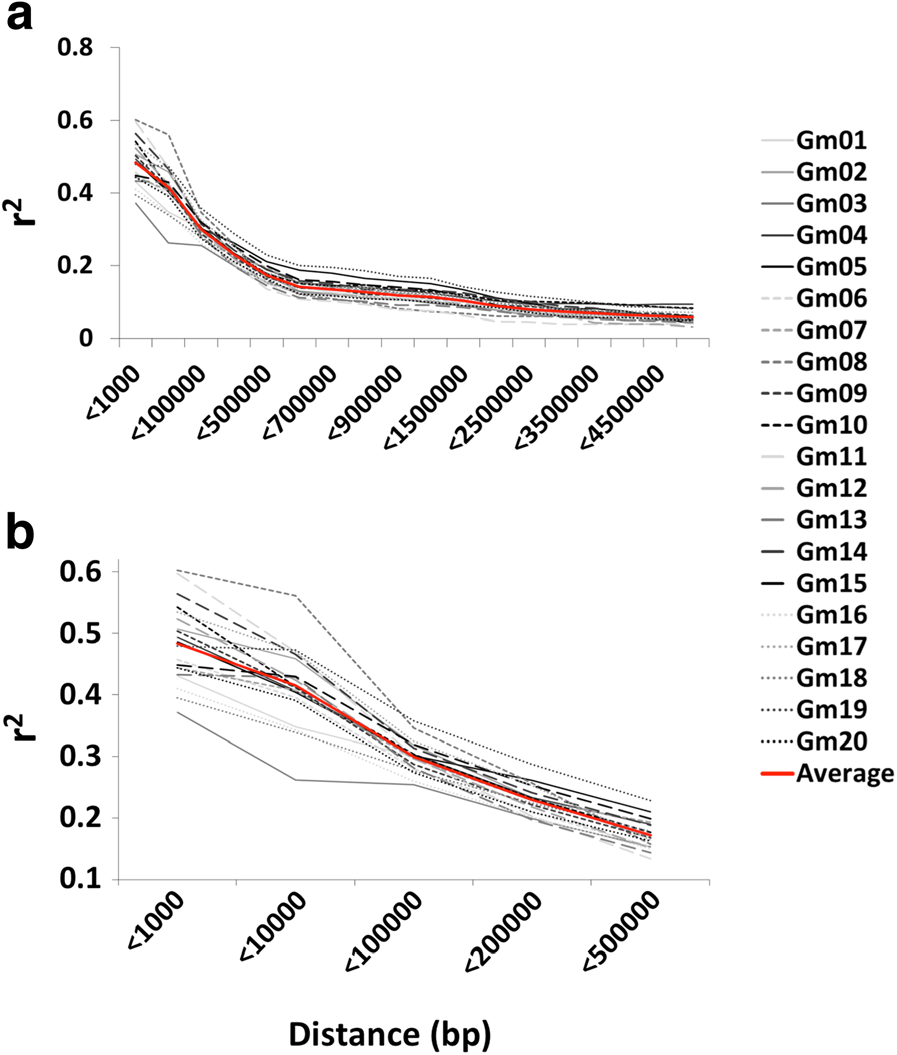
Linkage disequilibrium decay plots across soybean chromosomes and the average decay across the genome. **a** Average LD between SNPs with a maximum distance of 5 Mb. **b** Zoom-in of average LD between SNPs with a maximum distance of 500 kb

Linkage disequilibrium decay rates varied across the soybean chromosomes, with the average 50% decay rate occurring between 150 and 200 kb. Exceptions were with chromosome 11, which hit its 50% decay mark at ~ 100 kb, and chromosomes 5, 15, 18 and 19, which had slower decay rates of approximately 325 kb (Fig. 4). The average starting linkage disequilibrium rate for SNPs within 100 bp was 0.48 and reached 50% of this value at approximately 180 kb.

### Genome-wide association analysis of soybean traits

Genome-wide association analysis was performed for soybean agronomic traits of interest across two environments for up to 3 years. After removing SNPs in 100% LD and with MAF < 0.05, a total of 14,594 SNPs, 13,364 SNPs and 31,283 SNPs were selected for GWA analysis in the GBS, SoySNP50K microarray and merged data sets, respectively. To limit the effect of false positives in the analysis, population structure (covariate *Q*) or principle components (covariate *P*) were incorporated into the model as covariates. In the first approach, GWA analysis was performed using GLM analysis incorporating *P* or *Q* as covariates, while the second approach utilized a kinship matrix (covariate *K*) using CMLM analysis incorporating *P* or *Q* as covariates for a total of seven different models being performed on each trait to choose the optimal model for the population and given trait (Additional file 6). Models incorporating kinship and covariates *P* (CMLM) often resulted in model underestimation, while incorporation of only *P* or *Q* typically led to model overestimation. As such, the model incorporating only covariate *Q* (i.e. GLM with three populations) was used. The models for each trait were then adjusted with Bonferroni multiple corrections to reduce false positives, and further validated using pairwise comparisons between significant SNPs.

**Fig. 5:**
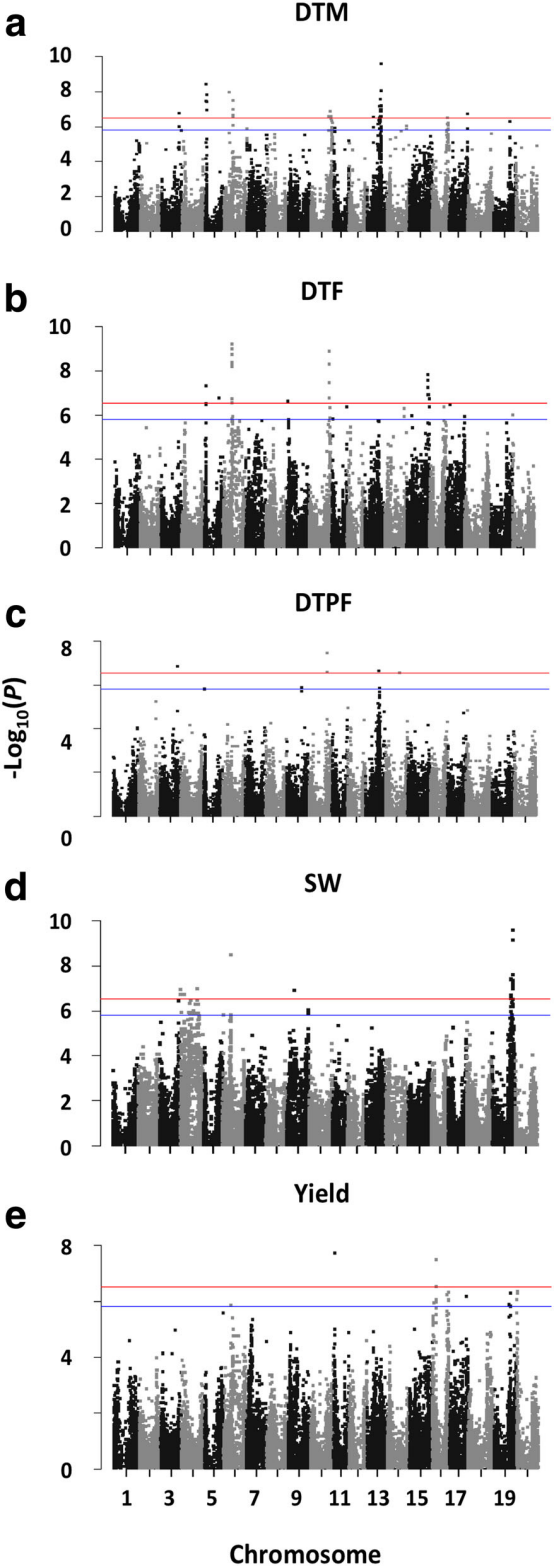
Genome-wide association analysis Manhattan plots for (**a**) days to maturity (DTM), (**b**) days to flowering (DTF), (**c**) days to pod filling (DTPF), (**d**) 100 seed weight (SW), and (**e**) yield. Lines represent the significance threshold as determined by Bonferroni multiple comparisons corrections equivalent to *P* < 0.05 (blue lower line) or *P* < 0.01 (red upper line)

GWA analysis with the different data sets was performed for soybean days-to-flowering (DTF), days-to-maturity (DTM), days-to-pod-filling (DTPF), yield and 100 seed weight (SW). Loci detected as significant when analyzing the GBS or SoySNP50K microarray data sets alone were similar, but less comprehensive than those reported when analyzing the merged data (Table 2; Additional file 7). In general, loci detected as significantly affecting a trait in the GBS or SoySNP50K microarray analyses were detected in the merged GWA analyses; however, not all loci detected in the merged data set were present in the GBS or SoySNP50K microarray results (Table 2). Exceptions were for 100 seed weight and yield, where significant loci covered larger regions or were not detected in the merged data set (Additional file 8), with the latter typically occurring in loci that were just below the significance threshold in their respective data sets. In the merged data set, a total of 74 SNPs located on seven different chromosomes were found to be highly associated (Bonferroni-corrected *P* value < 0.01) with DTM (Fig. 5a). Of these, six loci are not reported in SoyBase or recent literature and are potentially novel maturity loci (Table 2). Association analysis with DTF revealed similar results with 41 SNPs located on five chromosomes (Fig. 5b), while DTPF resulted in six SNPs on four chromosomes (Fig. 5c), 100 seed weight contained 41 SNPs on four chromosomes (Fig. 5d), and three SNPs on two chromosomes for yield (Fig. 5e). Potentially novel loci were also detected for DTM, DTF and DTPF (Table 2); however, no novel loci were detected for 100 seed weight or yield. Of the SNPs that were significantly associated with a trait, eight were found to be pleiotropic for DTM and DTF and one for DTM and DTPF. No SNP detected as significantly associated with 100 seed weight was detected in the other traits. Comparison of traits with differing SNP marker haplotypes using Tukey’s HSD test confirmed the significance of all markers detected below the Bonferroni correction limit of *P* < 0.01 (data not shown).

**Table 2:** Significant loci associated with important agronomic traits identified using genome-wide association analyses (Bonferroni correction *P* < 0.01)

Trait	Chr.^a^	MSS^b^ *P* value	Total SNPs^c^	Region		Average diff.^d^	Data set^e^	Novel loci^f^	Known genes/QTL^g^	Ref.^h^
				Start	End					
Days to maturity	3	1.82E-07	1	38,602,824	38,602,824	2.9	M,G	*		
	5	4.14E-09	12	1,927,907	3,263,938	7.2	M,G	*		
	6	1.08E-08	20	11,386,388	20,263,848	6.0	M,G		*E1*	[10]
	10	1.37E-07	4	40,769,008	46,434,446	3.1	M		*E2*	[9]
	13	2.90E-07	4	15,226,603	15,278,116	1.6	M,G	*		
	13	8.90E-09	14	29,022,554	31,262,263	3.8	M	*		
	13	2.67E-10	16	31,354,945	32,109,291	4.4	M,S		20-1	SoyBase
	16	3.08E-07	1	34,285,082	34,285,082	3.1	M	*		
	17	1.99E-07	2	40,939,386	40,970,920	5.6	M	*		
Days to flowering	5	5.11E-08	1	3,131,408	3,131,408	2.4	M,G,S	*		
	5	1.82E-07	1	33,211,040	33,211,040	4.2	M	*		
	6	6.35E-10	21	19,919,551	20,263,848	3.5	M,G		*E1*	[10]
	9	2.42E-07	1	3,031,973	3,031,973	6.0	M		24-2	SoyBase
	10	1.35E-09	8	46,241,807	46,580,047	2.9	M		24-4	SoyBase
	15	1.59E-08	9	48,460,246	51,379,618	4.1	M,G	*		
Days to pod filling	3	1.48E-07	1	38,579,331	38,579,331	2.9	M,G	*		
	10	3.62E-08	3	40,769,008	40,793,025	1.0	M,S	*		
	13	2.33E-07	1	29,387,862	29,387,862	8.3	M,G,S		7-2	SoyBase
	14	2.83E-07	1	31,326,567	31,326,567	1.0	M,S	*		
100 seed weight	4	1.12E-07	4	635,354	8,191,897	1.6	M		2-1; 6-2; 6-7; 13-4; 38-2; 47-3	SoyBase
	4	1.08E-07	1	37,659,105	37,659,105	1.6	M		36-15	SoyBase
	6	3.51E-09	2	18,315,510	18,446,052	2.1	M		15-1; 16-1; 16-2; 19-1; 34-16	SoyBase
	9	1.26E-07	1	17,708,693	17,708,693	1.7	M		30-5	SoyBase
	19	2.65E-10	33	40,130,037	43,116,996	1.8	M		5-1; 15-7; 17-1; 34-7; 35-7; 36-7	SoyBase
Yield	11	1.90E-08	1	2,584,048	2,584,048	574	M		2-1	SoyBase
	16	3.40E-08	2	7,914,714	7,985,838	67	M		23-13; 29-2; 31-9; 32-4	SoyBase

^a^Chr., chromosome number

^b^MSS, most significant SNP

^c^Total SNPs and regions including SNPs in 100% linkage disequilibrium with significant SNPs at Bonferroni correction *P* < 0.01

^d^Average difference in number of days to maturity, flowering, pod filling, 100 seed weight (mg) or yield (kg ha^−1^) between the different haplotypes of the most significant SNP (MSS) within the locus

^e^Data set(s) in which the significant locus was detected. M, merged data set; G, genotyping-by-sequencing data set; M, SoySNP50K microarray data set

^f^Loci not reported in SoyBase.org or recent literature for pod maturity (R8 full maturity), first flower, reproductive stage length (days to pod filling), seed weight or seed yield. Loci lacking stars represent known or previously reported loci, some of which genes are known and identified in the “Known Genes” column. Not all known loci have had associated genes identified

^g^Loci with known and identified genes or QTL previously reported as associated with the trait of interest

^h^References referring to genes or QTL previously identified. SoyBase refers to QTLs reported in the SoyBase database (www.soybase.org)

Among the SNPs detected as significantly associated with DTM and DTF, four were within 31.1 kb of *E1,* while one SNP was detected within 1 Mb of *E2* for DTM*.* No other SNPs were found within or around other known soybean *E* genes for any trait. Interestingly, a total of 14 SNPs significantly associated with DTM were found between 29.0 and 31.3 Mb of Gm13, a region that has not been reported as associated with maturity.

## Discussion

Genome-wide association studies are conducted to identify genetic loci associated with traits of interest [45]. This knowledge can then be utilized in breeding programs using marker-assisted breeding approaches. Although genotyping-by-sequencing is commonly used and has been shown to be reliable and efficient, this study shows that additional information obtained from other sources, such as microarrays, can help improve the depth of information obtained in GWA studies. Although intra-accession variation may exist within agricultural crop lines, the high concordance rate (98.8%) between overlapping SNPs between the GBS and SoySNP50K microarray data sets suggests that merging of two genotyping methods can be appropriate, particularly in self-pollinating plants such as soybean.

Population structure can greatly affect the statistical power during GWA analysis and as such several methods were tested to diminish false negative associations. In the present study, seven different statistical models were tested to determine the most appropriate model for the given data set (Additional file 6). Models utilizing population structure without kinship proved to result in the most appropriate distribution of *P* values compared to expected *P* values and was therefore chosen for GWA analyses. Population structure analysis showed that populations were highly similar to results obtained using principal component analysis and cladistics analysis (Fig. 3). The three reported populations could be tightly correlated with the country of origin and can be grouped into cultivars originating historically from Japan, China or a mix of unknown origins. Using the three main populations (GLM Q3) in the GWA analyses followed by adjustment of the significance threshold with Bonferroni multiple corrections resulted in a model providing SNPs that were significantly associated with and known to affect the various traits studied.

Inbreeding and selection can have large effects on linkage disequilibrium [18, 46]. As such, it has been estimated that the number of markers required to identify loci significantly affecting traits is in the tens of thousands for soybean [47]. In this study, a sufficient amount of markers (> 67,000) were obtained for detection of loci affecting various agronomic traits that have been highly selected for in breeding programs. Similar to other studies examining soybean LD [18, 20, 48], a diverse range of LD was observed across the various chromosomes (Fig. 4), with the highest LD occurring on chromosomes 19 and 5. As expected, chromosome 19 has been extensively utilized in soybean short-season breeding programs as it harbours the maturity locus *E3.* Chromosome 5, however, has been reported to affect maturity despite no known *E* genes being located on it [49–51]. The lowest levels of LD observed in this study were on chromosomes 8 and 11, with the former recently identified as harbouring the rare *E10* allele [11]. Aside from *E10* and possibly the as yet unidentified *E8,* no known maturity loci have been selected for on these chromosomes.

Complex traits such as maturity and flowering often make it difficult to assess the effectiveness of GWA analyses as they are often affected by multiple loci. Merging of GBS and microarray data not only resulted in a higher number of markers, but also proved to be complementary as not all regions detected as significant in one data set were detected in the other (Additional file 7). Additionally, merging of the data sets revealed loci not detected in either data set when analyzed alone, particularly for DTM (Additional file 7). A high number of significantly associated SNPs were observed for all traits, including days to maturity, a trait which has been highly selected for. Despite all of the lines being classified as 0 to 000, a high variation in the number of days to maturity (DTM), flowering (DTF) and pod filling (DTPF) was observed. Of the loci significantly associated with DTF, those on chromosomes 6 and 10 were also found to be significantly associated with DTM. This is not surprising as DTM and DTF were highly correlated (Table 1) and these two loci are known to have been highly selected for in early maturity breeding programs [9, 10]. Similarly, three of the four loci significantly associated with DTPF were also associated with DTM, two traits that were also highly correlated. Interestingly, no co-localization of loci was observed between 100 seed weight and the other traits despite a moderate correlation between DTF and seed weight. Therefore, selection for earlier maturing and high yielding varieties can be selected for independently of seed weight.

Of the known *E* genes affecting maturity, SNPs were only located in the region of *E1* on chromosome 6 for DTM and DTF, and *E2* for DTM (Table 2). This is in accordance with previous research demonstrating that *E1* is a major determinant of early maturity and flowering under field conditions [52, 53]. Interestingly, another SNP located on Gm06 was found to be within 174 kb of *GmWRKY76* (Glyma.06 g142000)*,* a soybean transcription factor recently shown to affect flowering when introduced into Arabidopsis plants [54], suggesting that this gene merits further investigation for its role in soybean maturity and flowering. Despite known allelic variation for *E3* [24] and *E4* (Tardivel, unpublished data) in this collection of PIs, no SNP was significantly associated with these loci, most likely due to even distribution of the haplotypes within each population (data not shown). Interestingly, a maturity locus on chromosome 16 was located within 3.1 Mb of the recently reported *E9* gene [55], suggesting that this chromosome could be further incorporated into soybean early maturity breeding programs. In addition to detecting loci associated with known alleles, several novel loci were detected for maturity traits (Table 2). These loci have most likely not been highly selected for and may show promise for utilization in early maturity breeding programs. As for 100 seed weight, no new loci were detected. Although novel loci were detected for DTM, DTF and DTPF, further work is required to fully validate the roles of these loci in controlling these traits.

Identification of genes involved in specific traits such as maturity is continuously advancing, as evidenced by recent publications identifying *E9* [55]*, E10* [11]*, J* [13] and *FT5a* [14]*,* as well as the possibility of *GmWRKY58* and *GmWRKY76* [54], which demonstrated roles in flowering in transgenic Arabidopsis. Several SNPs associated with DTM were found in proximity to several Arabidopsis *HAP5* (*NUCLEAR FACTOR Y* [*NF-Y*] family) orthologues, including *HAP5A* (Glyma.13 g207500), *HAP5B* (Glyma.13 g207700) and *HAP5C* (Glyma.13 g207600), and a *SNF2/EDA16* orthologue (Glyma.13 g183900). NF-Y proteins have been shown to help induce flowering, particularly under long days [56, 57]. These SNPs were also detected in the DTF and DTPF analysis at the Bonferroni correction level *P* < 0.05 level. The SNPs identified as significant on Gm05 for DTM were within 45,000 bp of Glyma.05 g036300, a gene encoding *SPERMIDINE/SPERMINE SYNTHASE*. *SPERMIDINE SYNTHASE* genes have been found to be essential for Arabidopsis embryo development and survival [58], and the close proximity of significant markers to this gene in soybean may indicate its potential role in soybean maturity and seed development.

For DTF, significantly associated SNPs were found within or near genes with a variety of biological roles, results similar to those detected for DTPF and 100 seed weight. Of particular interest for SNPs associated with DTF were SNPs in close proximity to the soybean Glyma.15 g275100, an orthologue to the human breast cancer gene *BRCA1* [59]. In Arabidopsis, this gene has been shown to be highly expressed in flower bud tissue [60], and is involved in homologous recombination and DNA repair [61]. For 100 seed weight, several SNPs were found in proximity to several *AUXIN RESPONSIVE PROTEIN* genes located on Gm04. Auxin has been shown to play a role in Arabidopsis embryo sac development as well as normal plant growth and development [62]. Several other SNPs associated with 100 seed weight SNPs on Gm19 were found within regions of a *DOWNSTREAM NEIGHBOR OF SON* (*DONSON*) or *HUMPTY DUMPTY* orthologue. DONSON proteins have been shown to be involved in DNA replication fork stability in humans [63], are required for ovary cell proliferation in Drosophila [64], and are linked with human microcephalic dwarfism [63]. The close proximity of SNPs associated with seed weight and the putative roles of *DONSON* in seed development and size suggest that this gene merits investigation for its role in plant seed development. Genes involved in carbohydrate metabolism, such as *BETA-FRUCTOFURANOSIDASE* and *ALPHA-AMYLASE* were also found to be in close proximity of SNPs associated with seed weight.

## Conclusions

The following study demonstrates that combining GBS and microarray data prior to performing GWA analyses can not only improve the power of detection, but also help identify loci that are overlooked by either method. This study also supports the concept that small population sizes that are genetically diverse can successfully identify loci governing traits of interest as known loci (*E1* and *E2*), as well as novel loci with Arabidopsis orthologues were identified. GWA analysis successfully identified loci with alleles previously known to affect the studied traits, but also novel loci containing genes orthologous to genes known to affect the traits in Arabidopsis. The GWA analyses have helped contribute to an enhanced understanding of known and novel genes affecting important soybean agronomical traits, which will be useful in future breeding programs utilizing marker-assisted selection. Further studies are currently ongoing to validate the roles of selected genes in soybean maturity.

## Additional files

Additional file 1: List of 86 lines used in the GWA analyses, along with their corresponding maturity group and fastStructure group. (XLSX 24 kb)

Additional file 2: Average trait values across the five site-years. (PPTX 87 kb)

Additional file 3: Quantile-quantile (QQ) plots of phenotypic traits demonstrating normal distributions. (PPTX 131 kb)

Additional file 4: Location of gaps and their distances in the genotyping-by-sequencing data set. (XLSX 11 kb)

Additional file 5: Location of gaps and their distances in the merged data set. (XLSX 12 kb)

Additional file 6: Quantile-quantile (QQ) plots of different genome-wide association study analytical approaches for various soybean agronomic traits. (PPTX 648 kb)

Additional file 7: Genome-wide association analysis Manhattan plots for soybean agronomic traits of interest using various genotyping methods. (PPTX 941 kb)

Additional file 8: Additional loci associated with important agronomic traits identified using genome-wide association analyses. (DOCX 14 kb)

## Abbreviations

CMLM: Compressed mixed linear model
*DONSON*: *DOWNSTREAM NEIGHBOR OF SON*
DTF: Days to flowering
DTM: Days to maturity
DTPF: Days to pod filling
*FT*: *FLOWERING LOCUS T*
GBS: Genotyping-by-sequencing
*GI*: *GIGANTEA*
GLM: General linear model
GWA: Genome-wide association
IBS: Identity-by-state
LD: Linkage disequilibrium
LML: Log marginal likelihood
MAF: Minor allele frequency
*NF-Y*: *NUCLEAR FACTOR Y*
PCA: Principal component analysis
*PHYA*: *PHYTOCHROME A*
PI: Plant introduction
SNP: Single nucleotide polymorphism
SW: 100 seed weight
