# Supplementary material for: Identification of novel loci associated with maturity and yield traits in early maturity soybean plant introduction lines
Source: BMC Genomics. 2018 Mar 1;19:167. doi: 10.1186/s12864-018-4558-4 (PMC5831853; doi:10.1186/s12864-018-4558-4)
Supplement: Supplementary file 2 — Average trait values across the five site-years. (PPTX 87 kb) [file 12864_2018_4558_MOESM2_ESM.pptx]

## Slide 1
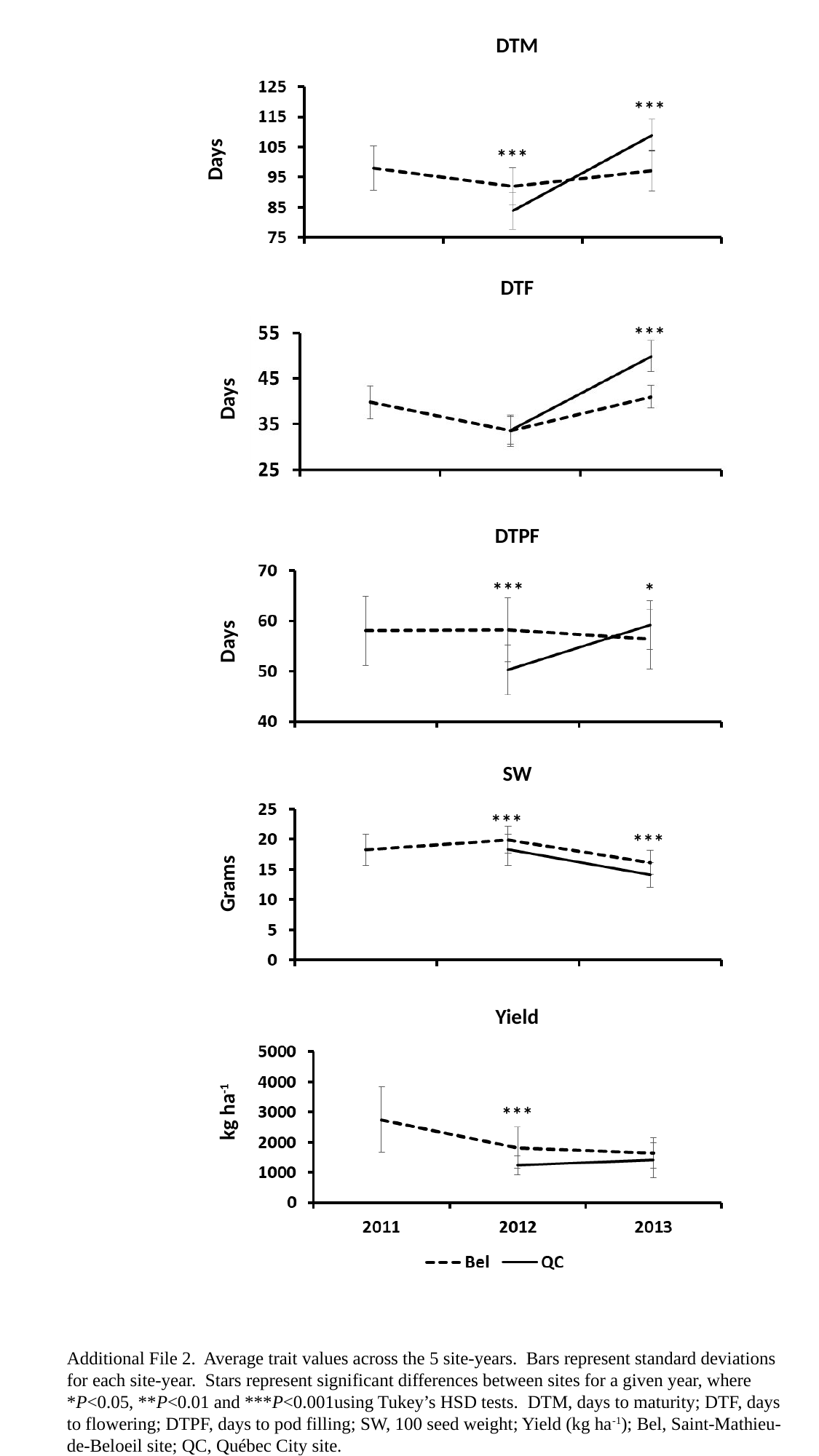

DTM
***
***
Days
DTF
***
Days
DTPF
***
*
Days
SW
***
***
Grams
Yield
kg ha-1
***
Additional File 2. Average trait values across the 5 site-years. Bars represent standard deviations for each site-year. Stars represent significant differences between sites for a given year, where *P<0.05, **P<0.01 and ***P<0.001using Tukey’s HSD tests. DTM, days to maturity; DTF, days to flowering; DTPF, days to pod filling; SW, 100 seed weight; Yield (kg ha-1); Bel, Saint-Mathieu-de-Beloeil site; QC, Québec City site.
