# Supplementary material for: Identification of novel loci associated with maturity and yield traits in early maturity soybean plant introduction lines
Source: BMC Genomics. 2018 Mar 1;19:167. doi: 10.1186/s12864-018-4558-4 (PMC5831853; doi:10.1186/s12864-018-4558-4)
Supplement: Supplementary file 3 — Quantile-quantile (QQ) plots of phenotypic traits demonstrating normal distributions. (PPTX 131 kb) [file 12864_2018_4558_MOESM3_ESM.pptx]

## Slide 1
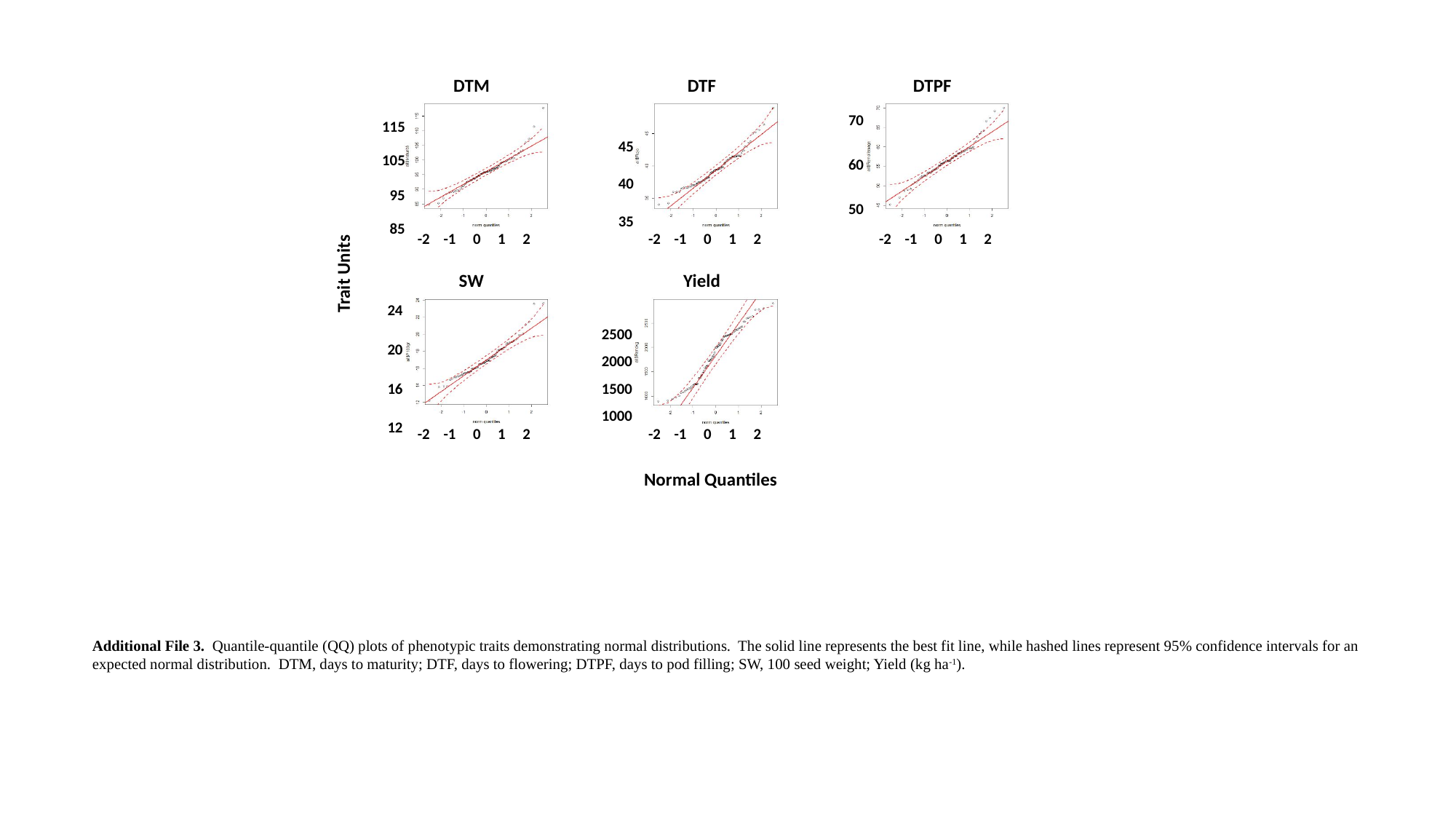

DTM
DTF
DTPF
70
60
50
115
105
95
85
45
40
35
-2 -1 0 1 2
-2 -1 0 1 2
-2 -1 0 1 2
Trait Units
SW
Yield
24
20
16
12
2500
2000
1500
1000
-2 -1 0 1 2
-2 -1 0 1 2
Normal Quantiles
Additional File 3. Quantile-quantile (QQ) plots of phenotypic traits demonstrating normal distributions. The solid line represents the best fit line, while hashed lines represent 95% confidence intervals for an expected normal distribution. DTM, days to maturity; DTF, days to flowering; DTPF, days to pod filling; SW, 100 seed weight; Yield (kg ha-1).
