# Supplementary material for: Identification of novel loci associated with maturity and yield traits in early maturity soybean plant introduction lines
Source: BMC Genomics. 2018 Mar 1;19:167. doi: 10.1186/s12864-018-4558-4 (PMC5831853; doi:10.1186/s12864-018-4558-4)
Supplement: Supplementary file 6 — Quantile-quantile (QQ) plots of different genome-wide association study analytical approaches for various soybean agronomic traits. (PPTX 648 kb) [file 12864_2018_4558_MOESM6_ESM.pptx]

## Slide 1
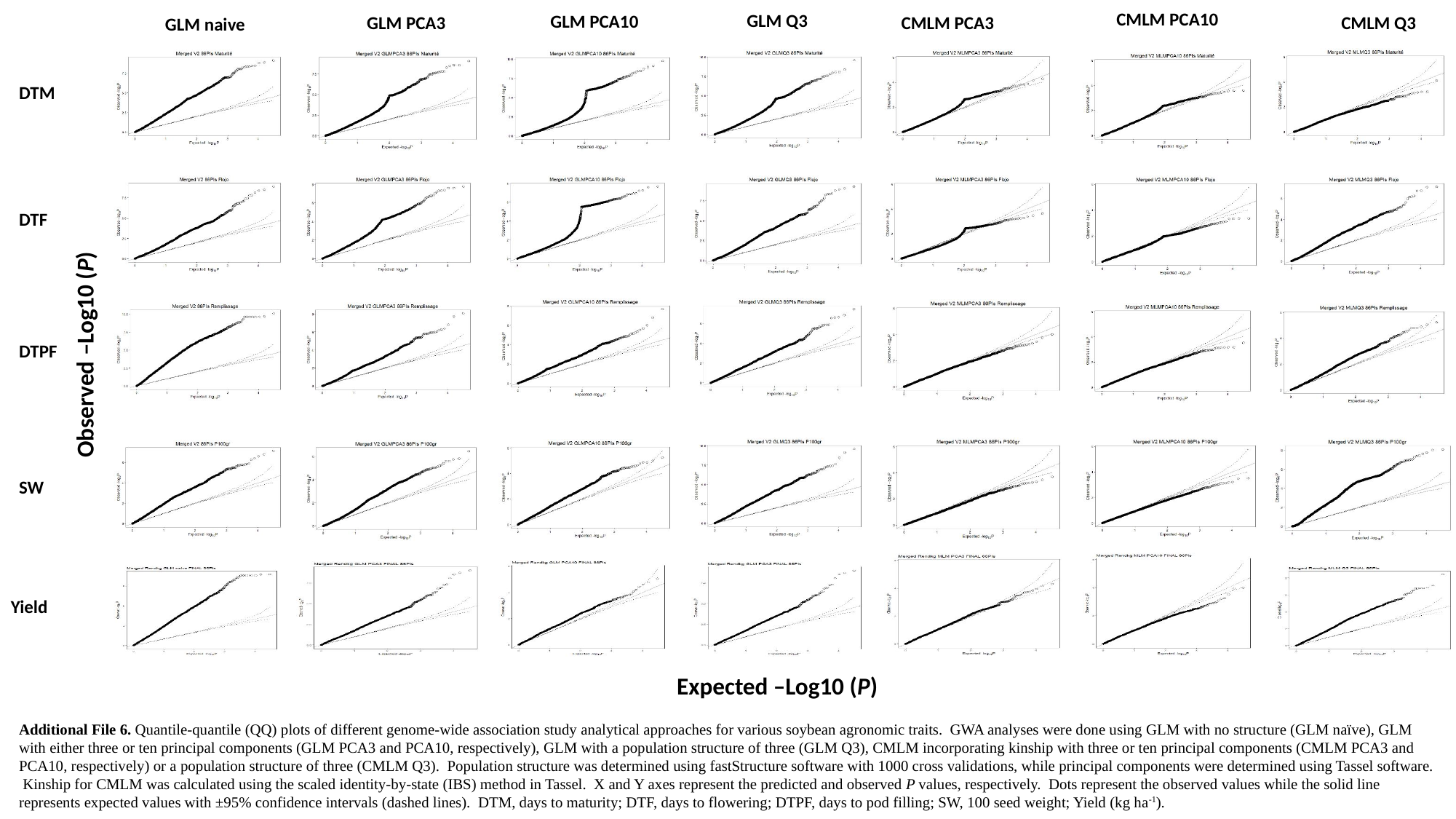

CMLM PCA10
GLM Q3
GLM PCA10
CMLM Q3
GLM PCA3
CMLM PCA3
GLM naive
DTM
DTF
Observed –Log10 (P)
DTPF
SW
Yield
Expected –Log10 (P)
Additional File 6. Quantile-quantile (QQ) plots of different genome-wide association study analytical approaches for various soybean agronomic traits. GWA analyses were done using GLM with no structure (GLM naïve), GLM with either three or ten principal components (GLM PCA3 and PCA10, respectively), GLM with a population structure of three (GLM Q3), CMLM incorporating kinship with three or ten principal components (CMLM PCA3 and PCA10, respectively) or a population structure of three (CMLM Q3). Population structure was determined using fastStructure software with 1000 cross validations, while principal components were determined using Tassel software. Kinship for CMLM was calculated using the scaled identity-by-state (IBS) method in Tassel. X and Y axes represent the predicted and observed P values, respectively. Dots represent the observed values while the solid line represents expected values with ±95% confidence intervals (dashed lines). DTM, days to maturity; DTF, days to flowering; DTPF, days to pod filling; SW, 100 seed weight; Yield (kg ha-1).
