# Supplementary material for: Identification of novel loci associated with maturity and yield traits in early maturity soybean plant introduction lines
Source: BMC Genomics. 2018 Mar 1;19:167. doi: 10.1186/s12864-018-4558-4 (PMC5831853; doi:10.1186/s12864-018-4558-4)
Supplement: Supplementary file 7 — Genome-wide association analysis Manhattan plots for soybean agronomic traits of interest using various genotyping methods. (PPTX 941 kb) [file 12864_2018_4558_MOESM7_ESM.pptx]

## Slide 1
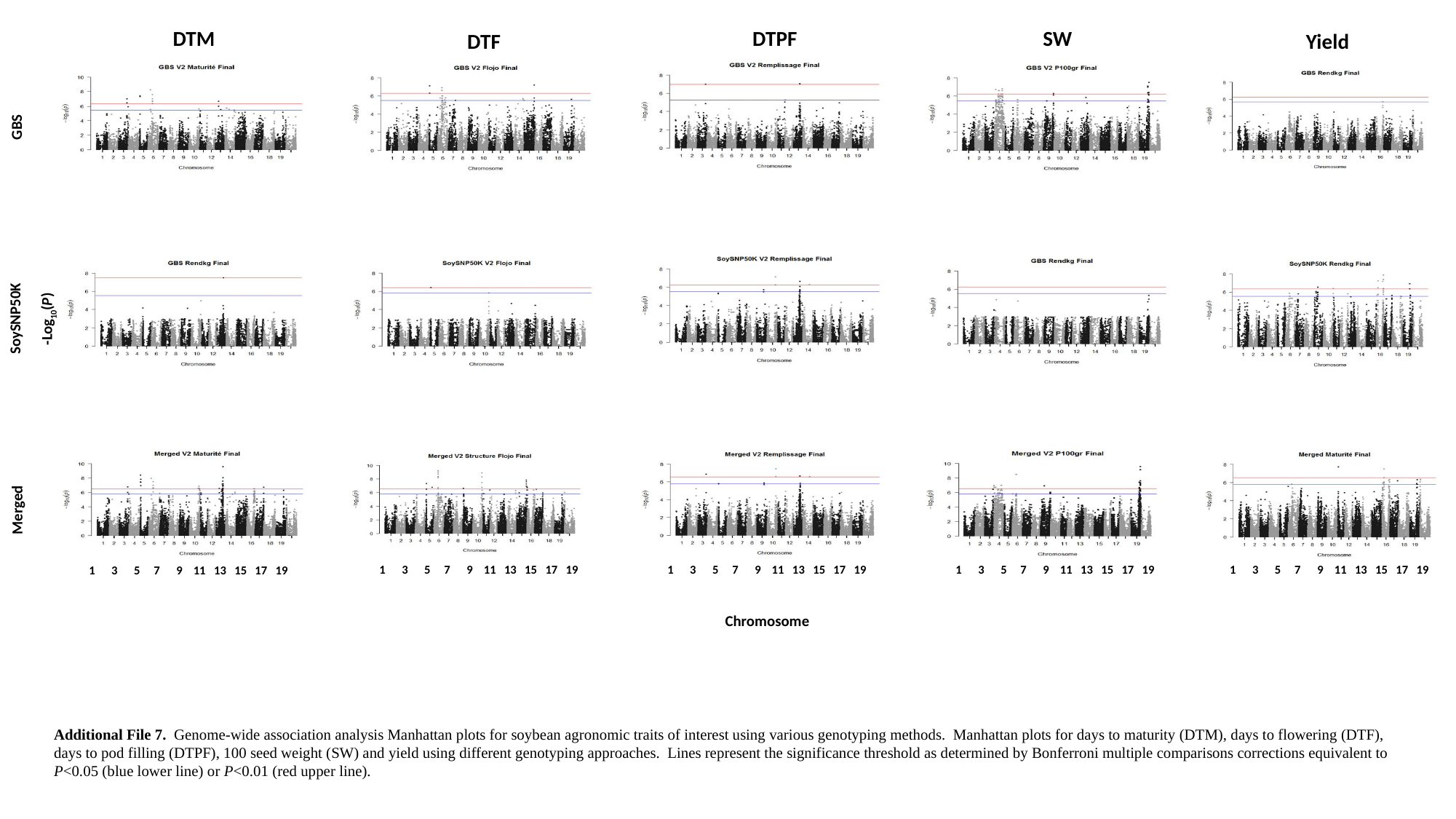

DTM
SW
DTPF
DTF
Yield
GBS
SoySNP50K
-Log10(P)
Merged
1 3 5 7 9 11 13 15 17 19
1 3 5 7 9 11 13 15 17 19
1 3 5 7 9 11 13 15 17 19
1 3 5 7 9 11 13 15 17 19
1 3 5 7 9 11 13 15 17 19
Chromosome
Additional File 7. Genome-wide association analysis Manhattan plots for soybean agronomic traits of interest using various genotyping methods. Manhattan plots for days to maturity (DTM), days to flowering (DTF), days to pod filling (DTPF), 100 seed weight (SW) and yield using different genotyping approaches. Lines represent the significance threshold as determined by Bonferroni multiple comparisons corrections equivalent to P<0.05 (blue lower line) or P<0.01 (red upper line).
