# Supplementary material for: Identification of novel loci associated with maturity and yield traits in early maturity soybean plant introduction lines
Source: BMC Genomics. 2018 Mar 1;19:167. doi: 10.1186/s12864-018-4558-4 (PMC5831853; doi:10.1186/s12864-018-4558-4)
Supplement: Supplementary file 8 — Additional loci associated with important agronomic traits identified using genome-wide association analyses. (DOCX 14 kb) [file 12864_2018_4558_MOESM8_ESM.docx]

**Additional File 8**. Additional loci associated with important agronomic traits identified using genome-wide association analyses

| **Trait** | **Chr.^A^** | | **MSS^B^ *P* value** | **Total SNPs^C^** | | **Region ^gg^** | | **Average diff.^D^** | **Data set^E^** | **Novel loci^F^** | **Known genes/QTL^G^** | **Ref.^H^** |
| --- | --- | --- | --- | --- | --- | --- | --- | --- | --- | --- | --- | --- |
|  |  |  |  |  |  | **Start** | **End** |  |  |  |  |  |
| **100 seed weight** | 4 | 2.71E-07 | | 4 | 21429333 | | 33248973 | 1.7 | G |  | 45-3 | SoyBase |
|  | 9 | 5.47E-07 | | 2 | 47291620 | | 47291622 | 1.4 | G | * |  |  |
| **Yield** | 9 | 2.97E-07 | | 1 | 21579839 | | 21579839 | 445 | S |  | 13-1 | SoyBase |
|  | 10 | 4.26E-07 | | 1 | 51501283 | | 51501283 | 466 | S | * |  |  |
|  | 16 | 1.38E-08 | | 5 | 32507199 | | 36750444 | 694 | S |  | 23-3; 21-5 | SoyBase |
|  | 19 | 1.30E-07 | | 2 | 34015909 | | 34038285 | 740 | S | * |  |  |

^A^Chr., chromosome number

^B^MSS, most significant SNP based on Bonferroni correction *P*<0.01

^B^Total SNPs and regions including SNPs in 100% linkage disequilibrium with significant SNPs at Bonferroni correction *P*<0.01

^D^Average difference in number of days to maturity, flowering, pod filling, 100 seed weight (mg) or yield (kg ha^-1^) between the different haplotypes of the most significant SNP (MSS) within the locus

^E^Data set(s) in which the significant locus was detected. M, merged data set; G, genotyping-by-sequencing data set; M, SoySNP50K microarray data set.

^F^Loci not reported in SoyBase.org or recent literature for seed weight or seed yield. Loci lacking stars represent known or previously reported loci, some of which genes are known and identified in the “Known Genes” column. Not all known loci have had associated genes identified.

^G^Loci with known and identified genes or QTL previously reported as associated with the trait of interest.

^H^References referring to genes or QTL previously identified. SoyBase refers to QTLs reported in the SoyBase database (www.soybase.org).s
